# Confronting Upside-Down Video-Assisted Thoracic Surgery Approach for Hemorrhagic Bronchogenic Cyst Manifested by Sudden Back Pain

**DOI:** 10.70352/scrj.cr.24-0126

**Published:** 2025-02-20

**Authors:** Masato Kambe, Tomonari Oki, Shuhei Iizuka, Yoshiro Otsuki, Toru Nakamura

**Affiliations:** 1 Department of General Thoracic Surgery, Seirei Hamamatsu General Hospital, Hamamatsu, Shizuoka, Japan; 2 Department of Pathology, Seirei Hamamatsu General Hospital, Hamamatsu, Shizuoka, Japan

**Keywords:** mediastinal neoplasms, bronchogenic cyst, thoracic surgery, video-assisted

## Abstract

**INTRODUCTION:**

Bronchogenic cysts, arising from an aberrant bronchial primordium inclusion during the fetal period, are typically located in the mediastinum but can develop in ectopic regions. While generally asymptomatic, these cysts may become symptomatic due to infection or, rarely, hemorrhage. This report details a case of a hemorrhagic bronchogenic cyst in the supradiaphragmatic region, successfully resected using video-assisted thoracic surgery (VATS) with a confronting upside-down monitor setting.

**CASE PRESENTATION:**

An 18-year-old female presented with a fever and sudden left-sided back pain. Blood tests revealed leukocytosis and an elevated C-reactive protein. Imaging studies identified a well-circumscribed cyst along the left diaphragm, suspected to be an infected bronchogenic cyst. Magnetic resonance imaging 2 days later indicated disease progression with concomitant empyema, prompting emergency surgery. Using the confronting upside-down monitor setting, the cyst was resected. Thoracoscopic findings revealed a dark red cyst and bloody pleural effusion. The surgery was uneventful, and the patient was discharged on postoperative day 2. Bacterial cultures of the pleural effusion and cystic content were negative, and histopathological analysis confirmed the diagnosis of a hemorrhagic bronchogenic cyst.

**CONCLUSIONS:**

Hemorrhagic bronchogenic cysts should be considered in the differential diagnosis of intrathoracic cysts presenting with sudden pain. Upfront surgery is recommended for symptomatic bronchogenic cysts, irrespective of the location or etiology. VATS via the confronting upside-down monitor setting is the feasible option alongside the conventional approach.

## Abbreviations

VATS
: video-assisted thoracic surgery
CT
: computed tomography
MRI
: magnetic resonance imaging

## INTRODUCTION

Bronchogenic cysts arise from an aberrant inclusion of the bronchial primordium in ectopic tissue during the fetal period.^1)^ They usually are located in the mediastinum but can develop in the extra mediastinum area.^2–9)^ Most bronchogenic cysts remain asymptomatic but may become symptomatic when infected.^10,11)^ Hemorrhage, however, is a rare complication with only a few cases reported in the literature.^12–15)^ A prophylactic surgical resection is recommended even for asymptomatic bronchogenic cysts, primarily because a salvage surgery following infection can lead to significant surgical morbidity, and video-assisted thoracic surgery (VATS) via the conventional look-up monitor setting is the standard approach.^10,16–18)^ We herein report a case of a hemorrhagic bronchogenic cyst in the supradiaphragmatic region, manifested by sudden back pain, which was successfully resected via a confronting upside-down VATS monitor setting.

## CASE PRESENTATION

**Fig. 1:**
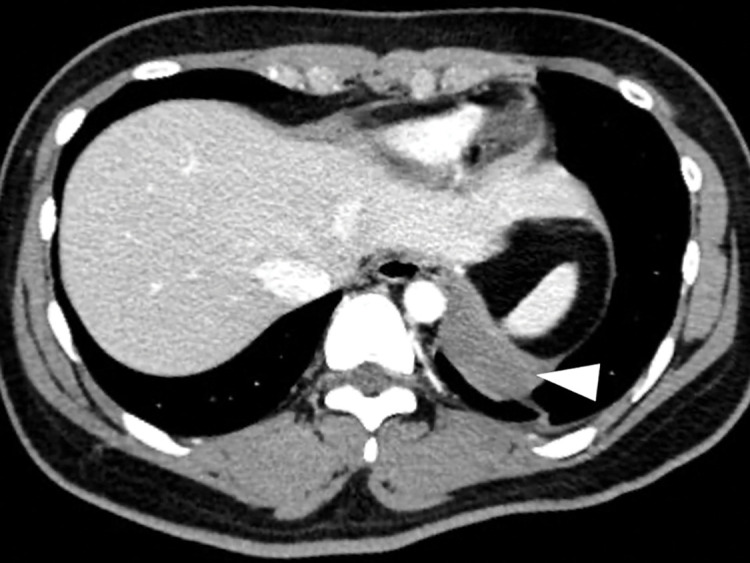
A chest CT showed a well-circumscribed mass (arrowhead) along the left diaphragm.

**Fig. 2:**
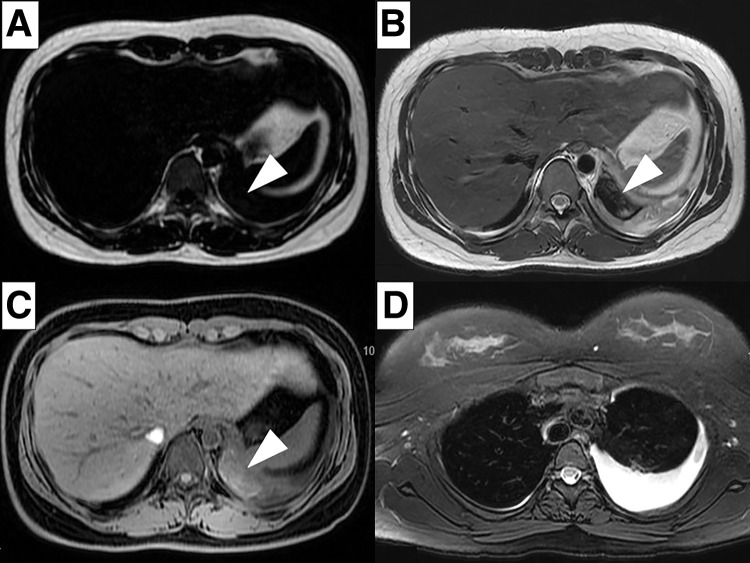
(**A**, **B**) MRI showing a low signal intensity both on the T1- and T2-weighted images (arrowheads). (**C**) Fat-suppressed T1-weighted images showing a slightly high signal intensity (arrowhead). (**D**) Fat-suppressed T2-weighted images showing fluid collection in the left pleural cavity.

An 18-year-old female presented with a fever and sudden left-sided back pain. She had no notable family or medical history and had been healthy until 2 days before the presentation. Blood tests showed leukocytosis (20810/μL) and elevated C-reactive protein levels (8.1 mg/dL), while hemoglobin levels (14.1 g/dL) were normal. Chest computed tomography (CT) revealed a well-circumscribed mass along the left diaphragm measured as 52 Hounsfield Units (HU) without contrast enhancement (**Fig. 1**). The maximum diameter was 40 mm. Based on the lesion’s cystic morphology and inflammatory features, an infectious bronchogenic cyst was suspected, and intravenous sulbactam sodium/ampicillin sodium was administered. Chest magnetic resonance imaging (MRI) 2 days later showed a low signal on the T1- and T2-weighted images and a slightly high signal on the fat-suppressed T1-weighted images, accompanied by a newly emerging left pleural effusion (**Fig. 2**). The preoperative blood tests showed persistent leukocytosis (19990/μL) and elevated C-reactive protein levels (28.68 mg/dL), while hemoglobin levels remained normal (13.0 g/dL). Those findings suggested a disease progression with concomitant empyema, promoting emergency surgery.

**Fig. 3:**
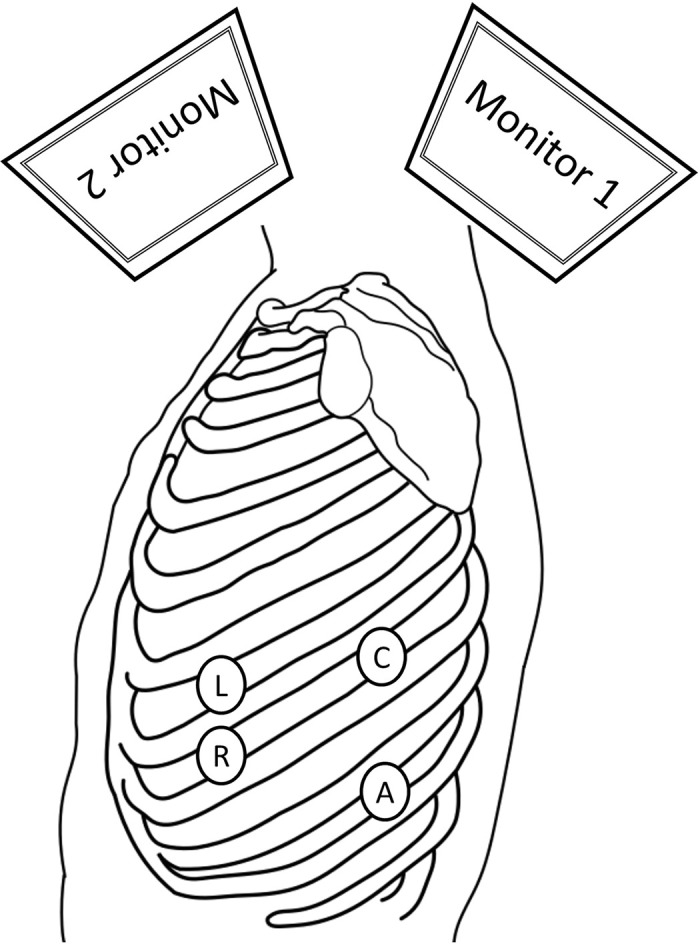
Port insertion sites: A 2-cm utility port for the surgeon’s right hand in the 7th ICS (R), 7-mm port for the surgeon’s left hand in the 6th ICS (L), 11-mm camera port in the 7th ICS (C), and 2-cm utility port for the assistant in the 9th ICS (A) were placed.

**Fig. 4:**
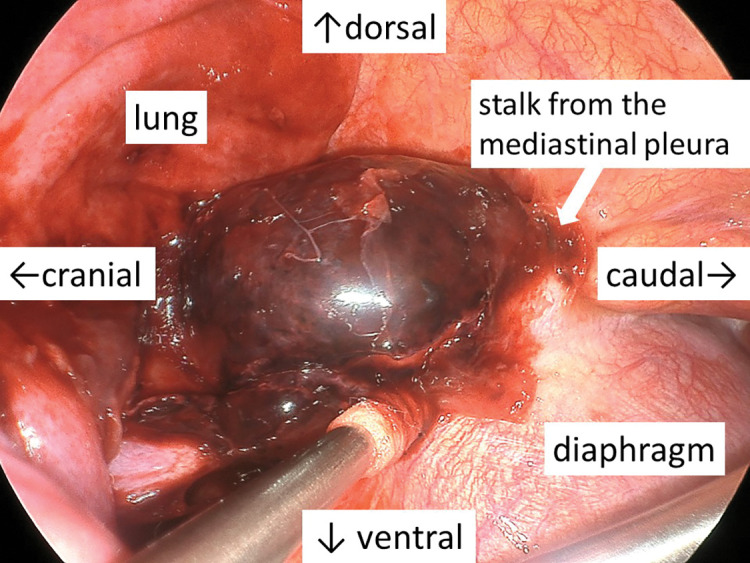
A dark red mass loosely adherent to the surrounding lung and diaphragm with a 2-mm width stalk from the mediastinal pleura.

**Fig. 5:**
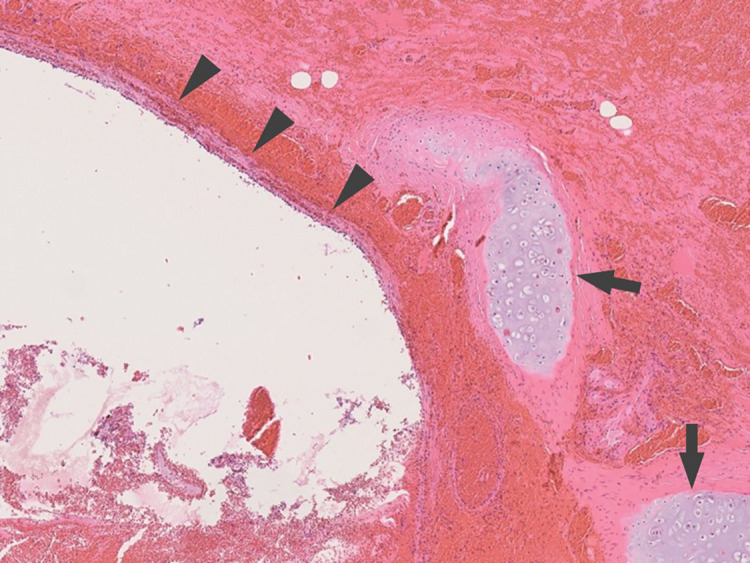
Histologically, an expanded bronchial structure (arrowheads) and cartilage tissue (arrows) are observed, with marked hemorrhage in the stroma.

The patient was positioned in the right lateral decubitus position under general anesthesia. The operator stood on the patient’s ventral side while the assistant and scopist stood dorsally. 2-cm utility ports were placed into the 7th intercostal space (ICS) on the anterior axillary line and into the 9th ICS on the posterior axillary line. An 11-mm camera port was inserted into the 7th ICS on the posterior axillary line, and a 7-mm port into the 6th ICS on the anterior axillary line. The 4-port VATS setup using a confronting upside-down monitor setting was established (**Fig. 3**).^19)^ The operator retracted the lung anteriorly using the sponge sticks to expose the mass while the assistant compressed the diaphragm caudally. The thoracoscopic findings revealed a dark red mass above the diaphragm and 380 mL of a bloody pleural effusion. The mass, loosely adherent to the surrounding lung and diaphragm, was pedunculated as a 2-mm width stalk from the mediastinal pleura (**Fig. 4**). It was resected along with the stalk. The source of the bleeding was not identifiable. The operation lasted 96 min, with a total blood loss (excluding pleural effusion) of 3 g. The patient was discharged uneventfully on the second postoperative day. Bacterial cultures of the pleural effusion and cystic content were negative. A histopathological examination revealed dilated bronchial structures and cartilage tissue, consistent with a diagnosis of a bronchogenic cyst (**Fig. 5**).

## DISCUSSION

The most common site of a bronchogenic cyst is the posterior mediastinum, followed by the lung parenchyma and other ectopic locations, such as the pleural or abdominal cavities, retroperitoneum, and cervical region.^4,5,20–24)^ While most bronchogenic cysts are asymptomatic and found incidentally, they can present with symptoms such as dyspnea, fever, or cough mainly due to an infection or compressive stress to the surrounding organs.^10,24)^ Hemorrhage is a less frequent initial manifestation.^18)^

We found 2 important clinical issues in the present case. Firstly, bronchogenic cysts can suddenly become symptomatic due to intracystic hemorrhage as well as infection, regardless of their location. Reported hemorrhagic cases have involved various locations, including the mediastinum, pleural cavity, and lung parenchyma, with lesion diameters ranging from 20 to 90 mm (**Table 1**).^12–15)^ Notably, all cases initially presented with sudden chest or back pain. The source of bleeding was identified in only one case involving the intercostal artery. Those findings suggest that bronchogenic cysts can become symptomatic suddenly due to intracystic hemorrhage, regardless of their location, size, or the etiology of the bleeding.

**Table 1:** Previously reported cases of hemorrhagic thoracic cysts

Case	Author [Reference]	Year	Age (years)	Sex	Symptoms	Located	Size (mm)
1	Desforges^12)^	1955	20	Female	Chest pain Dyspnea	Pleural Cavity	20
2	Muramatsu^13)^	2010	46	Male	Chest pain Dyspnea	Lung Parenchyma	Not available
3	Tsuzuku^14)^	2014	58	Male	Chest pain	Mediastinum	34
4	Suzuki^15)^	2020	34	Male	Back pain Dyspnea	Pleural Cavity	90
5	Kambe	2025	18	Female	Back pain Fever	Pleural Cavity	40

The present case also highlighted the diagnostic challenge of differentiating cystic infections and hemorrhages based on both clinical and radiological findings. Radiological features of bronchogenic cysts include a smooth, well-circumscribed morphology on CT and a low signal intensity on T1-weighted images with a high signal intensity on T2-weighted MRI. However, imaging characteristics can vary widely due to the difference in the cystic content viscosity, protein concentration, or hemorrhage.^25)^ These variations complicate the distinction between a cystic infection and hemorrhage based solely on the imaging findings.

In this case, an infectious bronchogenic cyst was initially suspected due to febrile symptoms, elevated inflammatory markers, and thoracic cyst characteristics consistent with prior reports. As the intraoperative finding of hemothorax and the negative pleural fluid culture ruled out empyema, it was considered reasonable to conclude that the symptoms were caused by the rupture of a bronchogenic cyst with intracystic hemorrhage. The cyst’s ectopic location further posed diagnostic confusion. Conservative treatment for infected bronchogenic cysts is often ineffective.^26,27)^ These findings underscore the importance of considering hemorrhagic bronchogenic cysts in the differential diagnosis of acutely symptomatic thoracic cysts and favor upfront surgery regardless of the cyst location or underlying etiology.

Secondly, VATS via a confronting upside-down approach is a feasible option for exploring the lower thoracic cavity area. Thoracoscopic surgery is the preferred treatment for bronchogenic cysts, with most cases employing the conventional look-up method.^16,28,29)^ However, in this case, the confronting upside-down method was chosen due to the lesion’s location near the aortic hiatus, just above the diaphragm. In the look-up monitor setting, the camera port placed in a lower intercostal space needs to approach from below the diaphragm, compressing its muscular portion to visualize the target. This can cause mirror-image confusion due to the monitor's head-side position. In contrast, the confronting upside-down setting allows direct access to the target from a higher ICS without diaphragm interference. Separate monitors, with one positioned upside-down, for the surgeon and assistant, eliminate the mirror-image confusion, enabling intuitive maneuvering akin to an open thoracotomy.^19)^

Additionally, this approach avoids the need to adjust the patient’s position based on the lesion’s location, a common requirement in the “look-up” method.^16)^ This may be considered another advantage, given that bronchogenic cysts can arise outside their typical site of occurrence.

## CONCLUSION

Intrathoracic cysts presenting with sudden back pain should include hemorrhagic bronchogenic cysts in the differential diagnosis. Upfront surgery is recommended for symptomatic bronchogenic cysts, regardless of their extra mediastinal origin and underlying etiology. VATS via the confronting upside-down monitor setting is the feasible option alongside the conventional approach.

## ACKNOWLEDGMENTS

We thank Mr. John Martin for proofreading the manuscript.

## DECLARATIONS

### Funding

The authors received no financial support for the preparation of this case report.

### Authors’ contributions

MK wrote this paper.

All authors read and approved the final manuscript.

### Availability of data and materials

Not applicable.

### Ethics approval and consent to participate

Not applicable.

### Consent for publication

Written informed consent was obtained from our patient for the publication of the case details.

### Competing interests

The authors declare that they have no conflicts of interest.
